# Predictors of Inappropriate Hospital Stay: Experience From Iran

**DOI:** 10.5539/gjhs.v7n3p82

**Published:** 2014-11-17

**Authors:** Ali asghar Ghods, Roghayeh Khabiri, Nayereh Raeisdana, Mehry Ansari, Nahid Hoshmand Motlagh, Malihe Sadeghi, Ehsan Zarei

**Affiliations:** 1 School of Nursing and Allied Health, Semnan University of Medical Sciences(SemUMS), Semnan, Iran; 2 National Institute of Health Research, Tehran University of Medical Sciences (TUMS), Tehran, Iran; 3 School of Public Health, Shahid Beheshti University of Medical Sciences (SBMU), Tehran, Iran

**Keywords:** appropriateness evaluation protocol, hospital stay, Iranian hospitals

## Abstract

**Objective:**

Hospital services are the most expensive component of modern health care systems and inappropriate hospital stay is one of the most important challenges facing hospitals in many countries. The purpose of this study was to determine the extent of inappropriate hospital stay and investigate the related factors in Semnan city (Iran).

**Methods:**

In this study, the Iranian version of Appropriateness Evaluation Protocol (AEP) was used in a representative sample of 300 hospital admissions and 905 hospital days. Data collection was performed during six weeks in January and February 2014 in four wards (two internal medicine and two surgical wards) of two hospitals in Semnan city (Iran).

**Results:**

The results showed that 7.4% of admissions and 22.1% of stays have been inappropriate. Inappropriate stays were mainly concerned to the factors, including length of stay, inappropriate admissions, as well as factors related to hospitals. The most frequent causes of unjustifiable days were due to waiting for diagnostic or therapeutic procedures (35.1%), and 20.6% delay in discharge of patients by physicians due to conservative medical policy.

**Conclusion:**

In conclusion, this study confirms the existence of inappropriate hospital stays which may be due to patient characteristics and hospital factors. The most unjustifiable reasons for inappropriate hospital stay were related to internal processes of hospital, which mostly could be prevented through appropriate management Therefore, some steps must be taken to decrease inappropriate hospital stay and preserve hospital resources for patients who need them.

## 1. Introduction

### 1.1 Introduce the Problem

Studies conducted in various countries indicated that the hospital care might be inappropriate and unnecessary in some cases and conditions (Campbell, 2001; Panis, Gooskens, Verheggen & Pop, 2003; Angelillo et al., 2000). Related studies have addressed both over–hospitalization and under–hospitalization through using the measurement of patient length of stay in hospitals. Their results indicated that in addition to clinical factors, non-clinical factors have also significant effects on the length of stay for patients with no complex conditions (Combes & Arespacochaga, 2013).

Iranian hospitals are usually funded by the public sector and they must cope with challenges of limited resources. They are usually crowded hospitals which seek to manage their limited recourses carefully and use them effectively (Joolaee, Tschudin, Nikbakht-Nasrabadi & Parsa-Yekta, 2008).

Meanwhile, avoiding inappropriate use of health care could lead to the saving of health care services costs without reducing the quality of these services. Most healthcare systems are actively seeking to reduce hospital beds (Green & Nguyen, 2001), They have opted this issue as a quality improvement process, which includes following steps: 1) the assessment of the inappropriate use of the services; 2) the determination of causes of the appropriate use; 3) the identification of wrong process; 4) the correction of wrong process and 5) finally the measurement of the effects of these changes (Matorass Galan, Depablo Casas, & Otero, 1990).

The emphasis of this study is mainly on the first and to some extent, on the second step of the above mentioned steps. The inappropriate use of hospital resources was examined through measuring two important processes in the hospital; inappropriate hospital admission and unnecessary hospital stay for patient in hospitals. These issues are considered as weaknesses of the health care systems in any country, which occur for various reasons and have considerable impact.

The results of the previous studies conducted in the Europe and United States have revealed that about 4 to 44.6% of the admissions were inappropriate and 4 to 48% of days of hospital stay were unnecessary (Angelilloet al., 2000; Panis, Verheggen & Pop, 2002; Barisonzo, Wiedermann, Unterhuber & Wiedermann, 2013; Vieira et al., 2006). The hospital beds occupied by patients with no indication for hospitalization and at the same time, there is no other non-hospital facility to transfer them there (“blocked beds”), are examples of the inappropriate stay in hospital. Although the term of “blocked bed” has no conventional definition and estimates of the prevalence of bed blockage vary and are largely subjective.

In this study, the inappropriate hospital admission refers to the admission of patients for whom there are other options with lower level of technology rather than the hospital services. In other words, there is no necessity to admit them in hospital at the present.

The appropriate hospital stay refers to the hospital stay for patients who need the continuous active medical, nursing and paramedical care provided in no other setting except hospital (Lavia & Anderson, 1996; Hartz, Bade, Sigmann, Guse, Epple, & Goldberg, 1996). The reduction of inappropriate stays (please consider *days of hospital stay*) in hospital could increase the hospital productivity and reduce the waiting lists, without endangering the quality of services.

On the other hand, according to the literature, inappropriate admissions and delayed discharges, which are relatively high, could result in unwanted consequences such as bed sores, hospital acquired infections, and consequently increasing healthcare costs (Hammond, Pinnington, & Phillips, 2009).

In scope of the management, the reduction of inappropriate days of hospital stays may result in cost savings and avoidance of wasting the resources. Besides, it provides a framework for continuous monitoring of hospital’s long term goals and objectives (Kossovsky, Chopardm et al., 2002).

## 2. Methods

### 2.1 Study Design

This study was a cross sectional analytical study that addressed hospital admissions and a prospective study which related to the hospital stay.

There are several methods to measure the inappropriate hospital admissions and stays and the AEP (Appropriateness Evaluation Protocol) is more common among them (Gertman & Restuccia, 1981). This study used the Iranian version of this protocol consisting of two tables. Table 1 includes criteria for appropriate admission of patients to hospital and Table 2 consists of three parts and generally 27 criteria to assess the appropriateness of each hospital day (11 criteria related to medical services, 7 nursing services, and 9 patient health status).

**Table 1:** Characteristics of study population

Variable		Hospital days		Total

		Appropriate(n=709)	Inappropriate (n=194)	
Sex	N(%)			
Male		368 (80.2)	91 (19.8)	459
Female		341 (76.8)	103(23.2)	444
Age(years)	N(%)			
≤40		341(86.1)	55(13.9)	396
>40		368(72.6)	139(17.4)	507
Marriage status	N(%)			
Single		245 (89.1)	30 (10.9)	275
Married		464 (73.9)	164 (16.1)	628
LOS	N(%)			
≤5		580 (79.9)	146 (20.1)	726
>5		129 (72.9)	48 (27.1)	177
Co morbidity				
No Co morbidity		429 (83.8)	83 (16.2)	512
Having Co morbidity		280 (71.6)	111 (18.4)	391
Insurance	N(%)			
No insurance		66 (91.7)	16 (8.3)	72
Having Insurance		643 (77.4)	188 (22.6)	831
living location	N(%)			
Urban		684 (79.0)	182 (21.0)	866
Rural		25 (67.6)	12 (32.4)	37

LOS, length of stay.

**Table 2:** Multiple Logistic regression model of inappropriateness of hospital stay

Variable	95% CL			p-value

	OR	Lower	Upper	
Hospital: Amir	3.69	1.29	10.54	.014
Sex: Male	.80	.384	1.66	.552

Age(years)	.99	.972	1.02	.759
Marriage status: Single	.61	.15	2.54	.504
Comorbidity: no co morbidity	.49	.145	1.66	.254
Urgent admission	1.25	.59	2.67	.550
LOS >5	4.39	1.50	12.82	.007
Insurance: No insurance	1.16	.28	4.78	.830
Inappropriate admission	2.98	1.32	7.54	.044
living region: Urban	2.67	.59	11.95	.198

OR, odds ratio; CI, Confidence interval, Nagelkerke R^2^: 0.202, Hosmer–Lemeshow: P=0.251, Corr. classification: 70.7%.

The protocol can be used for adult patients except psychiatric and labour and delivery patients (Restuccia, 1995).

The data collection was performed during six weeks in January and February 2014 that covered the male and female beds in four wards (two internal medicine and two surgical wards) in two selected hospitals in Semnan city (Iran).

### 2.2 Sampling Procedures

To conduct the study, in the first day, the interviewers visited different wards and received the list of patients who had been admitted to hospital on same day. Then some of the patients were randomly selected and information about appropriateness of admission according to Table 1 was completed for all of them using their records and clinical status. In the next days, the interviewers conducted the same process for the patients whom were investigated last days based on the criteria in Table 2 and for the newly admitted patients. Thus, in the subsequence daily visits to the hospitals, Table 2 was completed for the old patients and Table 1 was being completed for the new patients. The completion of the protocol was continued for each patient until his/her discharge or death. It should be noted that the interviewers ’visits to hospitals were on a daily basis and continued until the appropriateness evaluation protocol was completed for 300 patients. In case of the detection of an inappropriate stay for each day of hospitalization, the interviewer recorded its reason which was not included in AEP criteria. Overall a total of 905 hospital day stays was investigated in the study.

**Figure 1:**
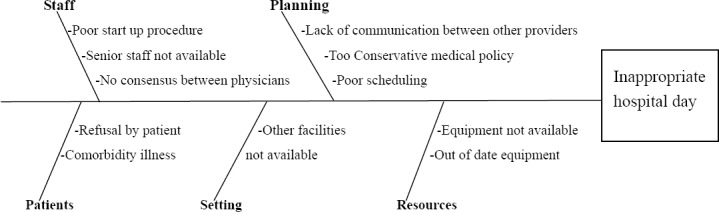
Ishikawa diagram on factors leading to inappropriate hospital day (Panis, Gooskens, Verheggen & Pop, 2003)

Causes of inappropriate hospital stay were classified into the seven main categories. The fishbone diagram was used for defining the potential causes of inappropriate hospital stay, including five categories of patient, staff, planning, setting and resources (Figure 1).

### 2.3 Data Analysis

We included the following variables that we considered likely to be related to the inappropriateness of hospital stay: patients’ sex, age, marital status (single, married), the duration of hospital stay (under 5 days, 5 days, and over 5 days), being insured, type of admissions (urgent or elective), and having co-morbidity, appropriateness of admissions and residential location (living in an urban or rural area).

Multiple logistic regression analysis (Hosmer & Lemesho, 2000) was performed to explore main effects and interactions among variables. A main effect is the effect of one of the independent variables on the dependent variable, ignoring the effects of all other independent variables. In other words, in a two way table, an independent variable could be found statistically significant; however, after controlling for other explanatory variables, it may not be significant anymore. In this study, the dichotomous variable ‘inappropriate stay’ was considered as the dependent variable.

To simplify interpretation of the findings, patient’s age and co-morbidity status were mean-centered (Tabachnick & Fidell, 2001), and length of stay (LOS) was categorized (0=<5 days’, 1=>5 days’), Type of admission was categorized (0=urgent’, 1= elective’) and appropriateness of admission was categorized (0= appropriate’, 1= inappropriate’)

Multi-co linearity among the independent variables was assessed by examining tolerance and the Variance Inflation Factor (VIF). All of the variance inflation factors values were quite satisfactory.

All analyses were intention-to-treat, and the effects are presented as odds ratios and 95% confidence intervals. The model’s goodness of fit was also tested using the Hosmer–Lemeshow test. A descriptive analysis was used for patients’ reasons for inappropriate stay.

All data analyzed using the SPSS software package, version 16.0. Statistical significance was set at p <0.05.

### 2.4 Ethics

This study was done in accordance to the Declaration of Helsinki, and was approved by the Ethics Committee of Semnan University of Medical Sciences (reference no: 92/372874). All participants received required information about the study and signed a written consent.

## 3. Results

A total of 903 days of hospital stay were evaluated. Findings showed that 49.7% of our sample were women and 51.3% were men with mean age of 42±27 years. Using the modified version of the AEP, 194 hospital days (21.5%) were rated as inappropriate. Rates of inappropriate patient stay according to patient characteristics are presented in Table 1.

As the logistic regression model shows (Table 2), LOS factor in patients’ staying longer than 5 days was strongly associated with an increased likelihood of inappropriateness of a hospital day [odds ratio (OR)=4.39, 95% confidence interval (CI)=1.50–12.82]. According to the Table 2 hospital variables was significantly associated with inappropriate hospital day, so in Kowsar hospital the inappropriate hospital day has been about four times more than that in Amir Hospital. [OR=3.69, 95% CI=1.29–10.54]. Also inappropriateness in admission increased the inappropriate hospital day about three times more than who have appropriate admission. [OR=2.98, 95%CI=1.32–7.54].

Also findings of logistic regression analysis showed that co-morbidity, age, sex, marital status, living area of patientsand having health insurance were not significantly related to the inappropriate hospital day.

Causes of inappropriate hospital stay days:

Causes of inappropriate hospital stay were classified into different main categories. The most frequent causes (35%) were related to delays in performing laboratory tests and clinical examination and (20%) delay in medical decisions (Table 3).

**Table 3:** Reasons for inappropriate hospital days of stay (n=194)

Reasons	days	Percent
Physician not available	7	3.6
Consultation or physician opinion	19	9.8
Postponement of surgery	9	4.6
Delay in therapy or diagnostics	68	35.1
Too conservative medical policy	40	20.6
Temporary discharge	6	3.1
Consultation and diagnostic test	23	11.9
Etc.	22	11.3

The rates of criteria explaining inappropriate hospital day of stay are presenting Table 3. Only 14.4% of inappropriate days were due to reasons unrelated to the acute care hospital; 85.6% of the unjustifiable days were due to hospital-related problems. For example, 35.1% of inappropriate days were due to patients waiting for performing diagnostic or therapeutic procedures or conservative decision making of some physicians about patient discharge (20.6%).

Based on the fishbone diagram (Figure 1), the delay in treatment or diagnostics and too conservative medical policy are especially related to ‘Planning’, whereas lack of availability of physician is categorized as caused by ‘staff’.

## 4. Discussion

This study was performed to determine the extent of inappropriate hospital stay in the internal and surgical wards of two hospitals in Seman city in the North Central of Iran.

An audit program was performed in order to confirm inappropriate hospital stay using the Iranian version of AEP. In addition, causes and risk factors for lengthened hospital stay were investigated.

Although the overall mean of length of stay in the hospitals of Seman city (3.02 days) is lower than the national average (2.7 days) (Khosravi, Khabiri, Aghamohammadi, & Najafi, 2012; Zarei, Daneshkohan, Pouragha Marzban, & Arab, 2015), inappropriate hospital stay rate in this study was 21.5%, which is in line with the findings of previous studies conducted in Iran (Hatam, Askarian, Sarikhani, & Ghaemm, 2010; Pourreza, Kavosi, Batebi, & Mahmoudi, 2006).

The highest inappropriate rates were reported by studies which only assessed the last day of hospital stay (Monteis Catot et al., 2006; Houghton, Bowling, Jones, & Clarke, 1996). The lowest inappropriate hospital stays were reported by cross-sectional surveys, which means that assessment of appropriateness hospital stay on a certain day is independent of the patient’s LOS (Panis, Verheggen, & Pop, 2002; Vieira et al., 2006; Kossovsky, Chopardm et al., 2002; Dizdar et al., 2007; Luis Zambrana Garcia et al., 2001).

This study identified several predictors of inappropriateness of hospital day. Patients hospitalized in Kowsar hospital were more likely to have inappropriate hospital days. In addition, the likelihood of inappropriateness has increased gradually during the hospital stay, regardless of its overall duration.

Given the multiplicity of possible causes of inappropriate admission and days, avoiding inappropriate hospital care is somehow difficult (Barisonzo, Wiedermann, Unterhuber, & Wiedermann, 2013). The likelihood of a hospital day being inappropriate may depend on patient characteristics, the organization of in-hospital care and the co-ordination between hospital care and the rest of the health care sector (Huet & Cauterman, 2005).

Overall, the inappropriate stay in hospital can be attributed to various external and internal factors. The external factors leading to the inappropriate stay in hospital are the factors out of the hospital control such as “blocked beds” On the other hand, the internal factors can be controlled by organization (hospital manager). These factors are mainly related to the inefficacy in medical care, the organization of patient as well as organizational problems (inefficiency in organization management).

According to our findings, the most unjustifiable reasons for inappropriate days of hospital stay were due to hospital-related problems i.e. delay in performing diagnostic or therapeutic procedures or delay in medical decision-making.

These factors are related to internal processes of hospitals and consequently could be controlled by the managers of hospitals (Panis, Verheggen, & Pop, 2002).

The results of our study showed that the mean length of inappropriate hospital stay was 1.74 days, which is in line with findings of the previous studies conducted in Iran (Zarei, Daneshkohan, Pouragha Marzban, & Arab, 2015; Hatam, Askarian, Sarikhani, & Ghaemm, 2010).

By eliminating inapproppriate hospital days one can achieve better efficiency (and not necessarity effectiveness) of a hospital and not necessarily the system. However, there are always many factors outside the control of organization, but it seems we can reduce the inappropriate hospital stay to a large extent with aim of appropriate criteria. Although, reducing the inappropriate hospital stay may needs setting and performing some monitoring protocols and teaching programs for staff cooperation with this programs, that in turn may need some cost and time in short term, but in long term have many positive effects such as decreasing the wait list of hospital admission and consequently more patients would be access to treatment (Paniset al., 2003).

In general, the number of inappropriate days of hospital stay and admissions can be reduced to a large extent through implementing quality improvement methods. However, it should be noted that the first step in every quality improvement process is the identification the problem and its extent (what we sought to measure in this study).

## 5. Limitations of the Study

- The accuracy of information on each criterion of the protocol
- The incompleteness of the patients’ medical records (e.g. failure to accurately record the initial and final diagnosis)
- The lack of cooperation of physicians and nursing staff
- Missing some cases due to the self discharge of patients prior to the completion of the protocol Bias in the completion the protocol

Because of the large number of diseases, assessing the inappropriateness of patient stay based on the medical diagnosis would be an unwieldy action. The AEP criteria are based on the use of inpatient facilities rather than the assessment of the nature of the illness. These results are yielded from medical diagnosis of our patients and as a result, are applicable across a wide range of diseases and specialties. In addition, in assessing inappropriateness of inpatient stay, it is difficult to examine each day of stay in details. Using AEP, the explicit and objective criteria based on usage of facilities, can simplify this process.

Considering the US criteria of appropriateness, it seems that the instrument itself requires customizing based on the condition of other countries (e.g. in relation to the threshold for body temperature for fever necessitating hospitalization, intramuscular injections, vital signs monitoring).

## 6. Conclusions

Findings from this study confirm that both doctor’s approach and hospital’s functional organization are still among the most frequent reasons for inappropriate hospital days.

Lengthened hospital stay further increases the possibility of additional days being inappropriate.

The issue of inappropriate hospital admissions and stays has been obvious in this study and to some extent, their reasons were considered. The awareness of such problem requires us to initiate next steps on the issue. It seems that some points should be considered which are including: identifying wrong processes causing the inappropriate hospital admissions and stays, correcting these process through quality improvement methods, proper training of medical and nursing staff in identifying the criteria of the appropriateness evaluation protocol, expanding the use of outpatient diagnostic and treatment services, establishing non-hospital treatment facilities in the country’s healthcare system.

Hospitals in Iran may perform the role of the main providers of services due to a systemic issue, which is a misbalanced health care system where there are no facilities that could provide alternative services to patients that could less resource wasteful. In such case, a process improvement in hospitals and audit of appropriateness of admissions may not (will not) be sufficient.
